# Productive Performance and Blood Biochemical Parameters of Dairy Cows Fed Different Levels of High*-*Protein Concentrate

**DOI:** 10.3389/fvets.2022.852240

**Published:** 2022-04-11

**Authors:** Nikolai Buryakov, Dmitrii Aleshin, Maria Buryakova, Anastasya Zaikina, Mohammed Nasr, Mohamed Nassan, Mohamed Fathala

**Affiliations:** ^1^Department of Animal Feeding, Institute of Animal Science and Biology, Federal State Budgetary Educational Institution of Higher Education ‘Russian State Agrarian University, Moscow Timiryazev Agricultural Academy', Moscow, Russia; ^2^Animal Wealth Development Department, Faculty of Veterinary Medicine, Zagazig University, Zagazig, Egypt; ^3^Department of Clinical Laboratory Sciences, Turabah University College, Taif University, Taif, Saudi Arabia; ^4^Animal Husbandry and Wealth Development Department, Faculty of Veterinary Medicine, Alexandria University, Alexandria, Egypt

**Keywords:** Ayrshire cows, amino acid, rations protein levels, biochemical parameters, lactation period

## Abstract

This study investigated the productive traits and some blood biochemical parameters of high-yielding Ayrshire dairy cows fed at different levels of Agro-Matic@LLC NGO, Russia (Agro-Matic (AM)) protein concentrate. A total of 45 high-yielding Ayrshire cows were selected and divided into three groups, each 15. The control group (0AM) fed the basal ration, while group two (1AM) and group three (2AM) fed a basal ration by replacing sunflower cake with different levels of AM (1 and 1.5 kg/head/day), respectively. Milk and blood samples were collected. The current results revealed that the ratio of rumen undegradable protein to rumen degradable protein during the period of lactation was significantly higher in the 1AM and 2AM compared with 0AM and represented (55.04, 62.14, and 41.73%), respectively. The 1AM had a beneficial effect on the digestibility of crude protein. Daily and whole fat-corrected milk (FCM 4 %) was significantly increased by 3 kg/day and 987 kg/entire lactation in 2AM when compared with 0AM, respectively. Blood total protein was significantly higher in the 1AM group (86.9 vs. 77.8 g/l) than the 0AM, while AM decreased urea concentration. Consequently, the inclusions of AM protein concentrate have a positive impact on increasing milk production and optimizing the rations in terms of the amount of non-digestible protein and the economic efficiency of milk production.

## Introduction

The primary target of any program of dairy cattle feeding is to attain gainful milk production. The basis of increasing the productivity of animals is the creation of a solid forage base and the organization of full feeding balanced concentrated ration in order to realize the genetic potential of milk productivity, health, and reproduction of animals (1, 2). In feeding highly productive animals, special consideration is given to plant protein origin, which is widely used to balance diets in terms of protein and essential amino acids (3–5). Fodder of plant origin represented a higher proportion of the diet when compared with food of animal origin. Protein in animal diet is the most expensive component that accounts for 35–55 % of the cost of the diet and its efficiency varies widely in ruminants (6–8).

Balancing the diets of highly productive cows based only on the amount of crude and digestible protein without taking into account the chemical composition and the degree of breakdown by the rumen microflora leads to high consumption of protein diet, metabolic disorders, decrease in productivity, and profitability of livestock production (9). Thus, it is better to evaluate the protein nutritional value of rations using the amount of degradable and non-degradable protein. Non-degradable protein has a significant effect on the number of amino acids that pass into the small intestine (10, 11). Feeding dairy cattle requires using concentrate feed with feed additives containing energy and protein sources, which increase the quality and quantity of milk with improved reproduction traits (12).

There are several studies that used protein of different origins with different degrees of breakdown in the rumen of dairy cows (13–17). Regarding the recent studies of using new protein feed in diets of dairy cows has a positive effect on productivity, nitrogen metabolism, and health and reproduction (8, 18). To our knowledge, there are a few studies that were carried out on the feeding and production performance of Ayrshire dairy cows. We hypothesized that the AM supplementation will replace the sunflower cake of cows, a diet with high-economic benefits. Consequently, this study was aimed to assess the impact of AM protein concentrate inclusion on the milk yield and production performance, nutrient digestibility, milk physicochemical indicators, and some blood biochemical parameters of Ayrshire dairy cows.

## Materials and Methods

### Characteristics of Objects and Conditions of Research

This study was carried out from March 2018 to 2021 in a farm belonging to Agricultural Production Cooperative ‘Plemzavod Maysky' in the Vologda region, Russia. The research was carried out in accordance with the methodology approved by the Scientific Council of the Department of Animal Feeding and Breeding the Russian State Agrarian University (Protocol No. 49 of 07.02.2018). In total, forty-five high-yielding Ayrshire dairy cows on the dry period (three weeks before parturition), with an average body weight of 550 kg in the third lactation season taking into account the origin, age, live weight, physiological state, and milk productivity of more than 8,000 kg of milk per lactation were selected and divided into 3 groups (each group 15). The animals were clinically healthy and kept in the same conditions of housing and management throughout the experiment (3 weeks before parturition until the end of the lactation period). Cows were milked three times per day for 305 days.

**Figure 1:**
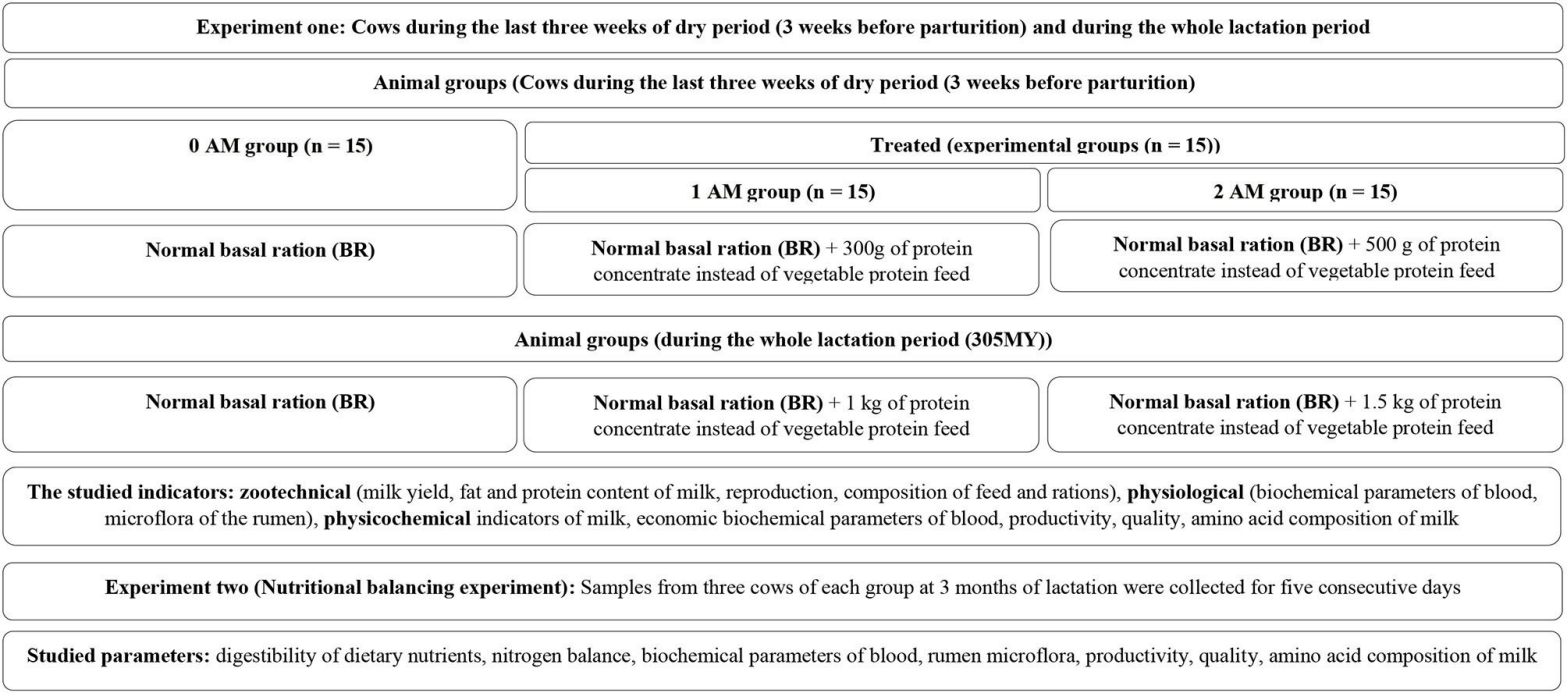
Experimental diagram.

The 0AM group received the normal basal ration after balancing the nutrient contents conferring to the high-yielding cows' requirements (All-Russia Institute of Animal Husbandry, VIZH, 2016) (Table 1). While, the 1AM and 2AM groups received normal basal ration replacing sunflower cake with 300 and 500 g of AM, respectively, until parturition. After parturition cows of 1AM and 2AM groups received normal basal ration replacing sunflower cake with 1 and 1.5 kg of AM, respectively, until the end of the lactation period (Figure 1). The Agro-Matic@LLC NGO, Russia (AM) consists of white lupine grain and poultry meat and bone meal (without feathers) (Tables 2–4). Amino acids in the AM were determined according to GOST 32195-2013 (19), while minerals except cobalt were measured by atomic absorption spectrometry in accordance with GOST 32343-2013 (20). Cobalt was determined by the photometric method in accordance with GOST 26573.2-2014 (21) in the testing center of the Federal State Budgetary Institution “VGNKI” (Moscow). The rations formulation was carried out using the Feed Optima program to fulfill the cows' requirements (energy, protein, vitamins, and minerals) during the dry period and lactation period. Actual daily milk yield, actual milk yield, 4 % FCM daily milk yield, 4 % FCM total milk yield, mass fraction of protein, fat and lactose content in milk and the non-essential amino acids in milk, physicochemical composition and quality indicators of milk, milk protein fractions were measured at the whole lactation period. The amino acids content in cow's milk was determined (22). Moreover, blood samples were collected from these cows at the end of the lactation period to estimate glucose, non-esterified fatty acids, ketone bodies, total protein, nitrogen content in the amino acids, urea, carotene, calcium, and phosphorus. Blood biochemical parameters were determined based on the certified laboratory of the BUVVO “Vologda Regional Veterinary Laboratory”. Total protein was measured by a refractometric method using IRV – 22 Refractometer with the help of The Reis table, but urea was estimated by the Michonne and Arnault method by the reaction with paradimethylaminobenzaldehyde. Spectrometric titration with the murexin indicator was used to measure the calcium level, while a colorimetric method based on the reduction of phosphoric-molybdenum acid was used to determine phosphorus level. Colorimetric method, color reaction with orthotoluidine reagent (Hultman method in Hyvarinen Nickel modification) was used to assess glucose. But the calorimetric gasoline extraction was used to evaluate carotene.

**Table 1:** Basal ration composition and the nutritional value of the rations of cows during the experimental period.

**Ingredients**	**Experimental groups**		
	**0 AM**	**1 AM**	**2 AM**
Mixed grass hay (kg)	0.5	0.5	0.5
Corn silage (kg)	7	7	7
Mixed grass haylage from cereals (kg)	7	7	7
Barley grain haylage (kg)	12	12	12
Beet molasses (kg)	1.5	1.5	1.5
Beet pulp (dry) (kg)	1.5	1.5	1.5
Soybean cake (kg)	1.0	1.0	1.0
Sunflower cake (36% CP) (kg)	1.5	0.5	-
“*Agro-Matic”* protein concentrate (kg)	-	1.0	1.5
Concentrated feed compound (kg)	11	10.5	10.5
Protected (oil) bypass oil (kg)	0.3	0.3	0.3
Monocalcium phosphate (g)	130	130	130
Sodium chloride (g)	120	120	120
**Nutritional value of the rations of cows during the experimental period**			
Metabolizable energy, MJ	279.0	275.5	276.6
Dry matter (g/kg)	238	234	238
Crude protein (g)	4,074.3	4,213.9	4,325.1
Crude protein (%)	17.10	18.00	18.20
Digestible protein	3,000.2	3,163.7	3,274.9
Degradable protein	2,880.5	2,725.4	2,674.6
Undegradable protein	1,202.1	1,500.0	1,662.1
Crude fiber	4,001.4	3,790.85	3,699.35
Crude fat	935.8	907.3	902.9
Neutral detergent fiber (NDF)	8,698.3	8,216.4	8,281.6
Acid-detergent fiber (ADF)	4,531.2	4,375.2	4,313.2
Starch	5,420.4	5,240.6	5,236.4
Sugar	1,665.7	1,638.9	1,630.8
Calcium	164.2	175.7	183.0
Phosphorus	126.0	125.3	126.1
Magnesium	54.0	52.9	52.8
Potassium	265.2	252.6	247.7

*0AM group: received the normal basal ration (Control group); 1AM group: received normal basal ration replacing sunflower cake with 300 g of protein concentrate Agro-Matic until parturition and after parturition they received normal basal ration replacing sunflower cake with 1 kg of protein concentrate Agro-Matic until the end of the lactation period. 2AM group: received normal basal ration replacing sunflower cake with 500 g of protein concentrate Agro-Matic until parturition and after parturition they received normal basal ration replacing sunflower cake with 1.5 kg of protein concentrate Agro-Matic until the end of the lactation period*.

**Table 2:** Nutritional value of protein concentrate (Agro-Matic/1 kg).

**Indicators**	**Contents (g)**
Metabolic energy, MJ	13.0
Dry matter content	932
**Nutritional value per 1 kg of dry matter**
Crude protein	582.5
Degradable protein	186.4
Undegradable protein	396.1
Digestible protein	502.4
Crude fiber	27.0
Acid detergent fiber (ADF)	23.3
Neutral detergent fiber (NDF)	130.5
Starch	10.0
Sugar	30.0
Crude fat	106.3

**Table 3:** The amino acid compositions of the Agro-Matic protein concentrate.

**Amino acids**	**Amount (g/100g)**
Methionine	0.59
Cystine	0.42
Lysine	2.08
Threonine	1.38
Arginine	4.12
Isoleucine	1.26
Leucine	2.40
Valin	1.84
Histidine	0.67
Phenylalanine	1.46
Glycine	7.20
Serin	1.99
Proline	4.92
Alanin	4.08
Tryptophan	0.23
Aspartic acid	3.61
Glutamic acid	6.45

**Table 4:** The content of mineral elements in Agro-Matic protein concentrate.

**Macro elements**	**g/kg**	**Micro elements**	**mg/kg**
Sodium	6.0	Iron	799
Calcium	34.0	Copper	4.8
Phosphorus	10.3	Zinc	33
Potassium	3.7	Cobalt	0.3
Magnesium	1.2	Manganese	121
Sulfur	11.6	Molybdenum	0.9
		Iodine	3.38
		Selenium	1.0

At 120 days of lactation, representative samples (milk, feces, and urine) were obtained daily for five consecutive days (balance experiment) to study the chemical composition, digestibility, and average daily nitrogen balance. Samples chemical composition was carried out in the department of livestock technologies of the Yaroslavl NIIZhK—a branch of the Federal Research Center “VIK im. V.R. Williams” (Mikhailovsky settlement, Yaroslavl region) [Tomme (23)]. Milk samples were collected in one common container and stored in a refrigerator at 4°C. Technological and physicochemical parameters of milk were carried out in the laboratory of the Vologda Dairy factory (Vologda) in accordance with GOST R 52054-2003 (24).

### Statistical Analysis

Data were statistically analyzed using the SPSS program with one-way ANOVA, according to the following model:

Yij = μ + Gi + Eij

Yij: Is an observed value of the dependent variable; μ: is a constant common to all observations. Gi: Is an effect due to ith treatment; (1 = 0 AM), (2 = 1 AM), and (3 = 2 AM); Eij: A random deviation due to unexplained sources of variation. Percentage data were subjected to the arcsine value. Duncan's multiple range tests were used for multiple comparisons among means at (*P* < 0.05). The Kolmogorov-Smirnov's test was used to test the data's normal distribution.

## Results

### Nutrient Digestibility and Nitrogen Balance

**Figure 2:**
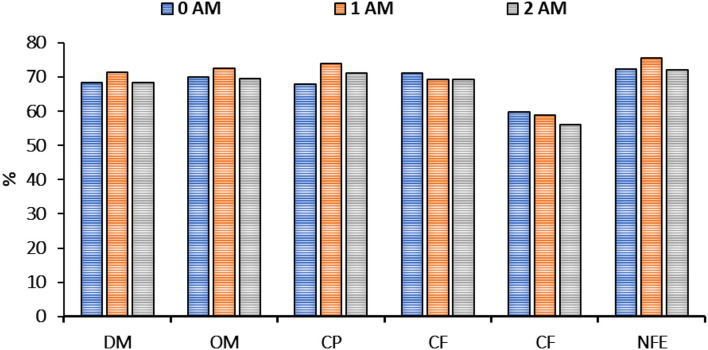
Coefficients of nutrients digestibility. 0AM group: received the normal basal ration (Control group); 1AM group: received normal basal ration replacing sunflower cake with 300 g of protein concentrate Agro-Matic until parturition and after parturition they received normal basal ration replacing Sunflower cake with 1 kg of protein concentrate Agro-Matic until the end of lactation period. 2AM group: received normal basal ration replacing sunflower cake with 500 g of protein concentrate Agro-Matic until parturition and after parturition they received normal basal ration replacing Sunflower cake with 1.5 kg of protein concentrate Agro-Matic until the end of the lactation period. DM, dry matter; OM, organic matter; CP, crude protein; CF, crude fat; CF, crude fiber; NFE, nitrogen-free extract.

The digestibility of organic matter in cows' diets ranged between 69.50 and 72.6% (Figure 2). Differences in the digestibility of crude fiber, crude fat, and nitrogen-free extract (NFE) among the cows of different groups were not significant (*P* > 0.05). The 1AM group revealed a beneficial effect on crude protein digestibility with digestibility of 73.9 (*P* < 0.05) vs. 68.0 % of the 0AM group (Table 5).

**Table 5:** Nitrogen balance in the lactating cows.

**Indicators (g)**	**0 AM**	**1 AM**	**2 AM**	**SEM**
Total feed intake	447.26^c^	495.20^b^	506.49^a^	9.20
Excreted with stool	143.58	140.54	148.29	1.98
Digestible	303.68^c^	352.66^a^	358.20^a^	8.82
Excreted with milk	161.18^b^	186.81^a^	196.67^a^	5.79
With urine	148.22^b^	165.09^a^	156.97^ab^	3.37
Remaining	155.49^b^	189.57^a^	201.23^a^	7.19
Balancing (±)	−5.69^b^	+2.76^a^	+4.56^a^	0.64

*0 AM group: received the normal basal ration (Control group); 1 AM group: received normal basal ration replacing sunflower cake with 1 kg of protein concentrate Agro-Matic until the end of lactation period; 2 AM group: received normal basal ration replacing sunflower cake with 1.5 kg of protein concentrate Agro-Matic until the end of the lactation period. SEM, Standard error mean*.

*Means within the same row with different superscripts were significant (P < 0.05)*.

**Figure 3:**
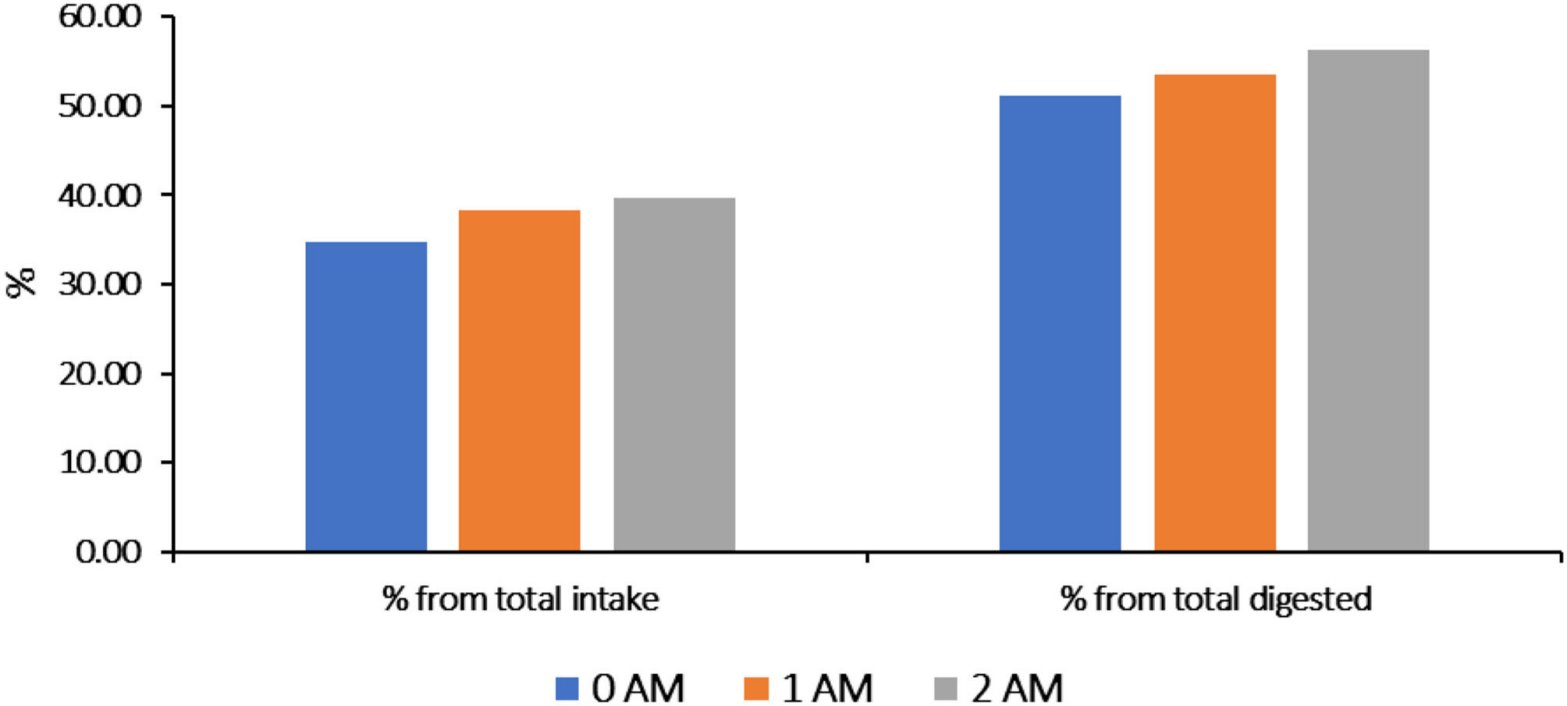
Nitrogen retention in the body of lactating cows (%). 0AM group: received the normal basal ration (Control group); 1AM group: received normal basal ration replacing sunflower cake with 300 g of protein concentrate Agro-Matic until parturition and after parturition they received normal basal ration replacing sunflower cake with 1 kg of protein concentrate Agro-Matic until the end of lactation period. 2AM group: received normal basal ration replacing Sunflower cake with 500 g of protein concentrate Agro-Matic until parturition and after parturition they received normal basal ration replacing Sunflower cake with 1.5 kg of protein concentrate Agro-Matic until the end of the lactation period.

The dairy cows excrete nitrogen (N) *via* milk, manure, and urine. Nitrogen metabolism is a set of complex transformation processes of protein, amino acids, and other nitrogen-containing substances in ruminants. There was high consumption of nitrogen (495.20 and 506.49 g, *P* < 0.05) with a positive nitrogen balance (+2.76 and +4.56 g) in the 1AM and 2AM groups, respectively. While nitrogen consumption was 447.26 g in the 0AM group with a negative nitrogen balance (−5.69 g). The highest nitrogen retention ratio was recorded in groups 1AM and 2AM (38.29 and 39.72 %) from the total feed intake with 53.46 and 56.19 % from the total digested protein, respectively. While in the 0AM group, it was 34.76 % from the total feed intake with 51.19 % from the total digested protein (Figure 3).

### Indicators of Productivity and Quality

The main indicator of the effective feeding of dairy cattle is milk yield with fat and protein content, which is based on the balanced diet. There was a significant effect of supplementing the diets with AM. It increased the daily milk yield of 4 % FCM with 3 kg/day in the experimental groups compared to the 0AM group. The total milk yield of 4 % FCM per lactation increased by 350 and 1,000 kg of group 1AM and 2AM, respectively, when compared with the 0AM group. The gross yield of milk fat and protein from the experimental groups was ~12.0 % higher (*P* < 0.05) than the control group (Table 6).

**Table 6:** The milk from cows (entire lactation season, 305 days) fed different levels of high protein concentrate (kg/cow).

**Parameters**	**0 AM**	**1 AM**	**2 AM**	**SEM**
Actual daily milk yield	28.9^b^	31.8^a^	31.4^a^	0.55
Actual total milk yield	8,442.2^b^	8,812.8^ab^	9,342.7^a^	150.08
Daily milk yield of 4% FCM	29.2^b^	32.2^a^	31.9^a^	0.53
Total milk yield of 4% FCM per lactation	8,512.4^b^	8,864.3^ab^	9,499.4^a^	161.15

*0 AM group: received the normal basal ration (Control group); 1 AM group: received normal basal ration replacing Sunflower cake with 300 g of protein concentrate Agro-Matic until parturition and after parturition they received normal basal ration replacing sunflower cake with 1 kg of protein concentrate Agro-Matic until the end of lactation period. 2 AM group: received normal basal ration replacing sunflower cake with 500 g of protein concentrate Agro-Matic until parturition and after parturition they received normal basal ration replacing sunflower cake with 1.5 kg of protein concentrate Agro-Matic until the end of lactation period. SEM, Standard error mean; FCM, fat corrected milk*.

*Means within the same row with different superscripts were significant (P < 0.05)*.

### Amino Acid Composition and Physicochemical Indicators of Milk

**Figure 4:**
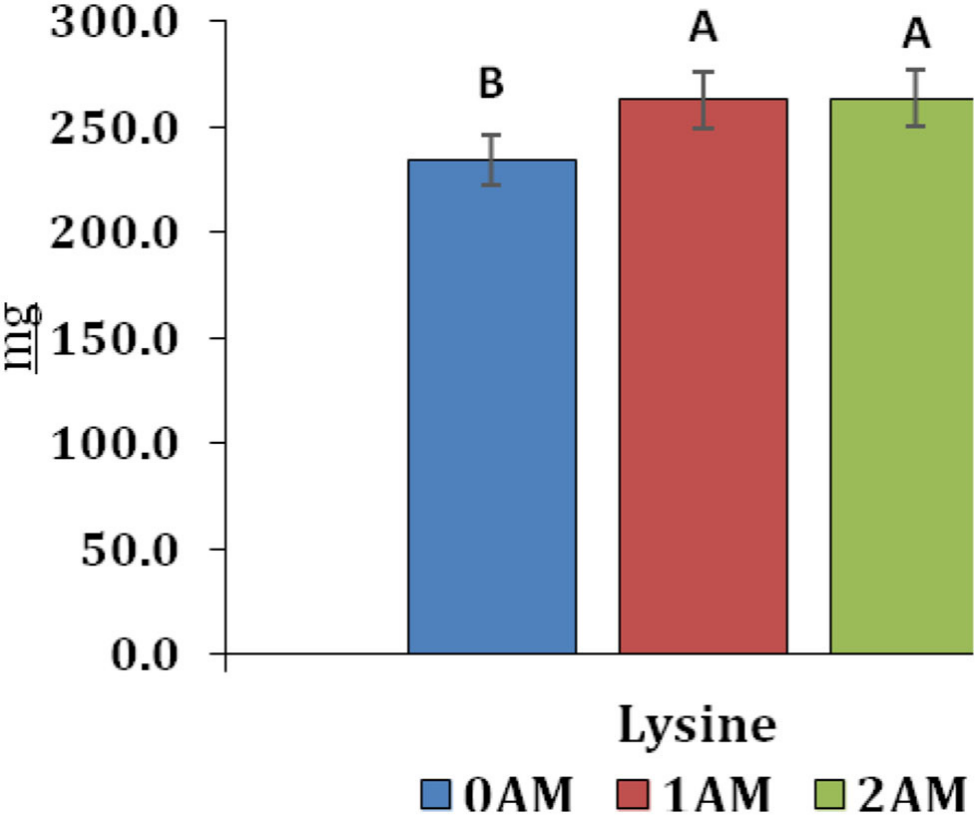
Lysine content of cow's milk at the end of lactation. 0AM group: received the normal basal ration (Control group); 1AM group: received normal basal ration replacing sunflower cake with 1 kg of protein concentrate Agro-Matic until the end of lactation period; 2AM group: received normal basal ration replacing sunflower cake with 1.5 kg of protein concentrate Agro-Matic until the end of the lactation period. Different letters revealed a significant difference (*P* < 0.05).

The AM increased the amino acids content of milk. There was 30 mg increase of lysine in groups supplemented with AM when compared with the 0AM group (Figure 4). The composition and nutritional value of the cow's milk influence their processing that determines the amount of the processed product and the economic efficiency. Regarding cheeses' production, milk quality must meet the requirements of state standards and Hazard Analysis and Critical Control Point safety principles. The density of milk depends on its components (proteins, fats, carbohydrates, and salts), and it was not significantly affected by AM supplementation (Table 7). Also, there were similar results of milk mass fractions among the three groups (Table 8).

**Table 7:** Physicochemical composition and quality indicators of milk of experimental cows during the period of lactation % (15 head/group).

**Indicators**	**Experimental groups**			**Requirements for milk (Top grade) GOST R 52054-2003 (22) GOST 31449-2013 (25)**
	**0 AM**	**1 AM**	**2 AM**	
Density, kg/m^3^	1,028.7	1,029.3	1,031.1	1,028.0
Titrated acidity, °T	16.0	16.0	16.0	From 16.0 to 21.0
Milk energy, kJ/kg	3,080.31	3,157.41	3,179.51	–
Mass fraction: fat	4.18	4.25	4.24	At least 2.8
Protein	3.22	3.34	3.41	At least 2.8
Lactose	4.35	4.47	4.52	–
DM %	12.25	12.42	12.68	–
Mass fraction of (DSMR)	8.52	8.89	8.92	At least 8.2
Thermal stability group	2	2	2	–
The content of somatic cells/cm^3^	<9 × 10^4^	<9 × 10^4^	<9 × 10^4^	No more 2.5 × 10^5^
**The cheese suitability of milk in terms of the ratio of nutrients**				**Standard**
Fat: Protein	1.30	1.27	1.24	1,10–1,25
Fat: (DSMR)	0.49	0.48	0.48	–
Protein: (DSMR)	0.38	0.38	0.38	0.35–0.45
Rennet-fermentation sample, class	3	1	2	At least 2
Cheese suitability	Bad	Good	Satisfactory	Good, Satisfactory

*0 AM group: received the normal basal ration (Control group); 1 AM group: received normal basal ration replacing sunflower cake with 1 kg of protein concentrate Agro-Matic until the end of lactation period; 2 AM group: received normal basal ration replacing sunflower cake with 1.5 kg of protein concentrate Agro-Matic until the end of the lactation period*.

**Table 8:** Content of milk protein fractions.

**Indicators**	**0 AM**	**1 AM**	**2 AM**	**SEM**
Mass fraction of whey proteins, %	0.89	0.89	0.98	0.05
Whey proteins, %	22.63	20.13	23.41	0.97
Mass fraction of casein, %	2.73	3.00	3.02	0.10
Mass fraction of urea, %	29.89	31.76	30.97	0.90
Mass fraction of NPN, %	0.020	0.016	0.019	0.001

*0 AM group: received the normal basal ration (Control group); 1 AM group: received normal basal ration replacing sunflower cake with 1 kg of protein concentrate Agro-Matic until the end of lactation period; 2 AM group: received normal basal ration replacing sunflower cake with 1.5 kg of protein concentrate Agro-Matic until the end of the lactation period. SEM, Standard error mean; NPN, non-protein nitrogen*.

### Biochemical Parameters

At the end of lactation, AM supplementation did not reveal any significant effect on the three groups (Table 9).

**Table 9:** Biochemical parameters of cows' blood at the end of the lactation period.

**Parameters**	**0 AM**	**1 AM**	**2 AM**	**SEM**
Glucose (mmol/l)	3.31	3.28	3.28	0.06
NEFA (mEq/ml)	0.39	0.37	0.37	0.02
Ketone bodies (mmol/l)	0.55	0.76	0.75	0.05
Total protein (g/l)	79.50	81.33	84.83	2.30
Nitrogen content in amino acids (mmol/l)	2.23	2.35	2.25	0.12
Urea (mmol/l)	3.59	3.51	3.15	0.16
Carotene (mg)	0.33	0.47	0.50	0.05
Calcium (mmol/l)	2.50	2.52	2.86	0.02
Phosphorus (mmol/l)	1.22	1.45	1.51	0.06

*0 AM group: received the normal basal ration (Control group); 1 AM group: received normal basal ration replacing sunflower cake with 1 kg of protein concentrate Agro-Matic until the end of lactation period; 2 AM group: received normal basal ration replacing sunflower cake with 1.5 kg of protein concentrate Agro-Matic until the end of the lactation period. SEM, Standard error mean*.

*Means within the same row with different superscripts were significant (P < 0.05)*.

## Discussion

Using protein of poultry processing raw materials will help to fulfill the shortage of plant protein by increasing milk production. Therefore, the current investigation was aimed to assess the impact of AM protein concentrate inclusion on the milk yield and production performance, nutrient digestibility, milk physicochemical indicators, and some blood biochemical parameters of Ayrshire dairy cows. The current results of the lactating cows' rations supplemented with AM had a positive effect on the digestibility coefficients of some nutrients compared with the 0AM group. These results were comparable with the findings of Paengkoum et al. (12), who reported that rations of growing Thai-indigenous beef cattle supplemented with high-non-degradable protein, improved nutrient digestibility. Also, this study showed that a high protein diet increased nitrogen excretion with feces, which was in accordance with others (26). They detected that total N and urea-N excretion in urine were greater for cows fed the high-CP diet compared with those fed the low-CP diet.

### Milk Production

Milk yield, fat, and protein content are more regulated by the balanced diets. Supplementation of AM increased cows' milk production. There were conflicting results about feeding a high-protein diet to the dairy cows. Some researchers stated that more dietary protein will increase the total milk production and 4% fat-corrected milk (27), while others did not detect any significant effect (26, 28). The physiological state of animals is the main factor providing high efficiency of obtaining high-quality milk, healthy offspring, and more longevity of cows. Milk quality influenced milk processing as cheese manufacture (29). For making cheese, milk must contain at least 3.1 % protein, more than 3.64 % fat with 125 mg calcium, 0.35:0.45 ratio of proteins and dry skimmed milk residue, and 1.10-1.25 fat-to-protein ratio. The density of milk for cheese preparation should be 1,027 kg/cm^3^, titrated acidity - 16–18°C (29).

The physicochemical composition of milk not only determines its nutritional and biological value but also affects all stages of processing. Regarding the technical regulations of the Customs Union 033/2013 on the safety of milk and dairy products, the content of DSMR is strictly regulated, since this indicator reflected the quality and usefulness of milk (29, 30). This study revealed that the content of DSMR value in cows supplemented with AM was higher than 8.5%, which corresponded to an “Extra” grade. Therefore, an unbalanced diet and poor-quality feed reduced milk quality and quantity, which consequently reduced cheese quality (30).

The milk composition is an important indicator for its processing and the economic efficiency of the dairy industry (31). Non-protein nitrogen of milk, including urea, has no biological value, so their high content can reduce the quality of the product (32). The use of AM with protein protected from a breakdown in the rumen reduces the yield of non-protein nitrogen in the milk of the experimental groups. In this case, the main role is played by casein, since it affects the structural and mechanical properties of the rennet, passing into cheese in the form of a para caseinate calcium phosphate complex (31).

### Biochemical Parameters of the Blood of Lactating Cows

Biochemical indices of blood are considered as an indicator of the health and physiological condition of animals. They reflect animals' metabolic processes. There was a significant effect of the AM supplementation on biochemical parameters. The total protein level was significantly increased due to the inclusion of AM. With reference to the content of free amino acids in the blood, it should be noted that during the period of lactation, their level was below the control level. This is primarily due to the high transition of amino acids in the milk of cows during this lactation period. At the end of lactation, the nitrogen level of free amino acids increased relative to the control group, and their highest content was observed in animals receiving 1.0 kg of AM as part of the daily rations of cows. Lysine was higher in the groups that were supplemented with AM. Lysine plays an important role in animal bodies. It contributes in collagen and elastin synthesis, hematopoiesis, synthesis of blood hemoglobin. It also improves calcium absorption, stimulates antibodies synthesis, and controls the conversion of carotene into vitamin A.

## Conclusion

Agro-Matic (AM) protein concentrate has been replaced with the sunflower cake in cows' diet. Its inclusion with different levels in the diet of highly producing Ayrshire dairy cows showed a positive influence on milk production during the entire lactation season, improved nutrient digestibility, nitrogen retention, and balance in the body without deterioration of cows' physiological and health status. Moreover, the inclusion of 1.5 kg/head/day was the best regarding economic benefits. Therefore, we recommended supplying AM in cows' diets for increasing milk production, improving the amount of non-digestible protein, increasing zootechnical and biological indicators of milk production.

## Data Availability Statement

The raw data supporting the conclusions of this article will be made available by the authors, without undue reservation.

## Ethics Statement

The research was carried out in accordance with the methodology approved by the scientific Council of the Department of Animal Feeding and Breeding of the Russian State Agrarian University (Protocol No. 49 of 07.02.2018).

## Author Contributions

NB, DA, MB, AZ, MNass, and MF: conceptualization, data curation, formal analysis, investigation, methodology, validation, visualization, roles and writing—original draft, and writing—review and editing. MNasr: formal analysis, roles and writing—original draft, and writing—review and editing. All authors contributed to the article and approved the submitted version.

## Funding

Taif University researchers supporting project number (TURSP-2020/71), Taif University, Taif, Saudi Arabia.

## Conflict of Interest

The authors declare that the research was conducted in the absence of any commercial or financial relationships that could be construed as a potential conflict of interest.

## Publisher's Note

All claims expressed in this article are solely those of the authors and do not necessarily represent those of their affiliated organizations, or those of the publisher, the editors and the reviewers. Any product that may be evaluated in this article, or claim that may be made by its manufacturer, is not guaranteed or endorsed by the publisher.
